# First Experience with [^18^F]FAZA PET Imaging in Kidney Allograft Dysfunction

**DOI:** 10.3390/diagnostics16162655

**Published:** 2026-08-20

**Authors:** Seyed Ali Mirshahvalad, Adam Farag, Shenghui Su, Olusegun Famure, Yanhong Li, S. Joseph Kim, Hong Sang Choi, Rohan John, Ana Konvalinka, Patrick Veit-Haibach

**Affiliations:** 1 University Medical Imaging Toronto (UMIT), Toronto Joint Department of Medical Imaging (JDMI), University Health Network (UHN), Sinai Health System & Women’s College Hospital, Department of Medical Imaging, University of Toronto, Toronto, ON M5G 2N2, Canada; adam.farag@uhn.ca (A.F.); patrick.veit-haibach@uhn.ca (P.V.-H.); 2 Department of Medical Imaging, Health Sciences North, Northern Ontario School of Medicine University, Sudbury, ON P3E 5J1, Canada; 3 Department of Medical Imaging, The Temerty Faculty of Medicine, University of Toronto, Toronto, ON M5S 1A8, Canada; 4 Toronto General Hospital Research Institute, University Health Network, Toronto, ON M5G 2C4, Canada; shenghui.su@uhn.ca (S.S.); ana.konvalinka@uhn.ca (A.K.); 5 Ajmera Transplant Centre, University Health Network, Toronto, ON M5G 2C4, Canada; 6 Division of Nephrology, Department of Medicine, University Health Network, Toronto, ON M5G 2C4, Canada; 7 Institute of Health Policy, Management and Evaluation, Dalla Lana School of Public Health, University of Toronto, Toronto, ON M5T 3M7, Canada; 8 Department of Laboratory Medicine and Pathobiology, University of Toronto, Toronto, ON M5S 1A8, Canada; 9 Institute of Medical Science, University of Toronto, Toronto, ON M5S 3H2, Canada

**Keywords:** nitroimidazole, kidney transplant, allograft rejection, microperfusion, volume distribution

## Abstract

**Objectives:** To evaluate ^18^F-labelled fluoroazomycinarabinoside ([^18^F]F-FAZA) PET in post-transplant patients and examine the association of its kinetic and uptake parameters with kidney allograft function measurements, including laboratory and histopathological assessments. **Methods:** In this prospective pilot study, renal transplant patients were evaluated with a dedicated positron emission tomography/magnetic resonance imaging (PET/MR) protocol between 2018 and 2023. Twenty patients with impaired kidney allograft function (cases) and ten sex- and time-post-transplant-matched control patients with stable transplant allograft function (controls) were included. All patients underwent [^18^F]F-FAZA PET/MR, including initial dynamic and delayed static imaging. Cases underwent an ultrasound-guided biopsy for histopathological assessments. **Results:** From the [^18^F]F-FAZA PET-derived parameters, dynamic total volume distribution (V_T_) was significantly higher in control patients than in cases (2.11 ± 0.55 vs. 1.62 ± 0.45; *p* = 0.026). When correlating [^18^F]F-FAZA PET parameters with creatinine levels and estimated glomerular filtration rate, V_T_ was again the only variable that significantly correlated with both metrics. Regarding histopathological parameters, a significant positive correlation was found between k_1_ and vascular fibrous intimal thickening, a significant negative correlation between k_3_ and arteriolar hyalinosis, and k_3_/k_4_ (binding potential) was significantly correlated with the percentage of globally sclerotic glomeruli and arteriolar hyalinosis. Additionally, V_T_ was significantly correlated with interstitial inflammation. SUV_mean_ on the static PET was significantly correlated with the percentage of globally sclerotic glomeruli. **Conclusions:** Non-invasive assessment of renal microcirculation using dynamic [^18^F]F-FAZA PET is potentially feasible. The dynamic PET-derived parameters showed strong correlations with renal function. There were also correlations with various histopathological findings. Additionally, static [^18^F]F-FAZA PET-derived parameters showed complementary findings with hypoxia-related end-stage histopathological indicators.

## 1. Introduction

Kidney transplantation is the treatment of choice for patients suffering from end-stage kidney disease (ESKD) [1]. Post-transplant premature kidney graft loss is a significant problem, resulting in increased morbidity, mortality and costs to the health care system. Although advanced immunosuppressive therapies have increased short-term kidney graft survival, long-term survival has only been partly extended over the past decades [1,2]. There is a wide range of immunological and non-immunological causes responsible for premature allograft loss, including antibody-mediated rejection, disease recurrence, de novo glomerulonephritis, non-specific development of fibrosis, infections, and drug-induced nephrotoxicity. Since transplant recipients are a very heterogeneous patient population with mixed clinical risk profiles and comorbidities, early identification of the underlying pathological process is critical to enable possible timely intervention, prior to the development of irreversible fibrosis.

Reduced kidney function is directly associated with poor graft and patient outcomes [1]. Monitoring of kidney health is routinely based on laboratory tests, including measured serum creatinine (Cr) levels, estimated glomerular filtration rate (eGFR), and assessment of proteinuria. Kidney transplant recipients may already have reduced kidney function compared to healthy individuals; thus, these parameters may be less reliable for detecting early kidney dysfunction, requiring intervention to prevent nephron loss [3]. Furthermore, current clinical evaluations are insensitive to subtle yet clinically significant changes in kidney function and lack specificity regarding the possible underlying mechanisms, necessitating confirmation through invasive kidney biopsy and histopathological assessment [4].

Mechanistically, kidney allograft interstitial fibrosis develops, at least in part, in the context of peritubular capillary dropout and progressive tissue hypoxia. Interestingly, peritubular capillary dropout is also a feature of antibody-mediated injury and ischemia–reperfusion injury. However, there are currently no clinical markers of kidney microvascular capillary dropout or microcirculation patency. There are various imaging modalities, such as US and MRI, to follow patients after kidney transplantation and visualize structural abnormalities in the allograft [5,6,7]. However, aside from gross abnormalities, such as hydronephrosis, or abnormalities in the arterial and venous flow, identification of microcirculation impairments and monitoring of microvascular lesions is not currently possible with these or other standard clinical imaging modalities.

^18^F-labelled fluoroazomycinarabinoside ([^18^F]F-FAZA) PET imaging has been used to detect and quantify hypoxia mostly in tumours [8,9]. To our knowledge, no previous clinical study has investigated the role of this PET agent in the context of kidney allograft dysfunction. Thus, the primary objective of this pilot project was to evaluate the feasibility of [^18^F]F-FAZA PET imaging in post-transplant patients with kidney allograft dysfunction and examine the association between its kinetic parameters and measures of kidney allograft function. As the secondary objective, we investigated the correlation between [^18^F]F-FAZA PET-derived parameters, as potential non-invasive functional measures of hypoxia-induced kidney allograft injury, and histological findings.

## 2. Materials and Methods

### 2.1. Patient Population

This institutional research ethics board-approved prospective pilot study evaluated kidney transplant recipients with a dedicated PET/MR protocol between November 2018 and March 2023. We included 20 patients with impaired kidney allograft function (cases), as evidenced by elevated serum Cr or significant proteinuria and a scheduled for-cause biopsy, and 10 sex- and time-post-transplant-matched control patients with stable kidney allograft function (controls). The matched control patients with stable kidney graft function (as defined by institutional guidelines) were identified by searching the kidney transplant registry (CoReTRIS) [10]. All participants signed an informed consent form prior to participating in this prospective study. Exclusion criteria included: (a) contraindications for MR as per current institutional guidelines; (b) inability to lie supine for at least 30 min; (c) any patient who was pregnant or breastfeeding; and (d) any patient unable or unwilling to provide informed consent.

For all patients, standard-of-care post-transplant investigations were performed, including clinical history, physical examination, blood work (e.g., complete blood count, electrolytes, serum Cr, liver function tests, calcineurin inhibitor levels), measurement of donor-specific antibodies, graft-specific Doppler ultrasound and assessment of proteinuria. As part of the routine care clinical pathway, only cases underwent an ultrasound-guided biopsy to identify the underlying cause of the graft impairment. Histopathologic findings were graded and interpreted as per clinical routine using the Banff scoring [11] by a renal pathologist (RJ). Clinical characteristics and biopsy features were correlated with tracer kinetic parameters calculated from the dynamic PET.

### 2.2. PET Acquisition

[^18^F]F-FAZA PET imaging was performed on a 3.0T PET/MR scanner (Biograph mMR Software Version VE11P, Siemens Healthineers, Erlangen, Germany). Patients were injected with a weight-based amount of furosemide (maximum of 10 mg) to ensure (as much as possible) that there was no overlying urine activity from the renal pelvis in the PET imaging. Prior to the PET/MR imaging, all participants (patients and controls) were asked to completely empty their bladder. Patients and controls were injected with up to 5.0 MBq/kg (maximum of 600 MBq) of [^18^F]F-FAZA on the PET/MR table. Then, dynamic PET acquisition began simultaneously with the injection and was acquired for the initial 20 min. The dynamic data were reconstructed as follows: 18 sets @ 5 s, 3 sets @ 10 s, 8 sets @ 30 s, and 7 sets @ 120 s (36 data sets overall). As part of the PET/MR protocol, MR Dixon-based T_1_-weighted imaging (T_1_-WI) was acquired simultaneously with the PET. Two hours after the initial injection, a static PET data set was acquired for 20 min. PET/MR acquisitions were performed at two bed positions: one in the abdomen covering the kidneys and the second covering the mediastinum to acquire images of background blood-pool activity for comparison.

### 2.3. Image Processing

Post-processed, reconstructed MR and PET image sets were transferred to dedicated image analysis software (PMOD Technologies Ltd., Zürich, Switzerland) and were accurately co-registered. All segmentation and modelling for this study were also performed using PMOD software (Version 4.4, PMOD Technologies LLC/Bruker, Zürich, Switzerland). The segmentation of the volumes of interest (VOIs) for the arterial input function (AIF) on dynamic PET was performed manually, being carefully done by two experienced PET readers in consensus in the aorta or proximal iliac artery supplying the transplant kidney. VOIs for the kidney were drawn on the peripheries of the two poles (upper pole and lower pole of each kidney) as well as the interpolar region and were contoured on T_1_W MR images to include only the kidney cortex. [^18^F]F-FAZA PET-imaging parameters were extracted from the upper and lower kidney poles since they were the sites of the biopsies. It is notable that the interpolar region was excluded, since (1) biopsies were not taken there, and (2) it is also anatomically close to the kidney hilum and therefore susceptible to inaccuracies from imaging spillover (to avoid a possible source of error in the modelling). Thus, as a first step, the average values of the upper and lower poles were used to follow a similar approach in all non-biopsied and biopsied patients. Later, to correlate with the histopathology findings, we focused on the region biopsied. The measured activities in the kidney were normalized to the activity in the blood pool of the mediastinum. On the static [^18^F]F-FAZA PET imaging (120 min after injection), the average standardized uptake value (SUV_mean_) was measured within the defined VOIs as well.

### 2.4. Kinetic Modelling

The image-derived time–activity curves (TACs) for each of the three VOIs in the kidneys and the AIF were decay-corrected and then used to perform the 2-tissue compartment (2TC) modelling with PMOD. The modelling provided the following kinetic parameters: $k_{1}$ (mL/cm^3^/min) as the rate constant of perfusion from the blood to the first tissue compartment, $k_{2}$ (min^−1^) as the rate constant representing the transport rate from the interstitium to the blood (washout of tracer back into blood), $k_{3}$ (min^−1^) as the tracer accumulation rate constant (phosphatization) in the second compartment (trapping, or specific binding rate), and $k_{4}$ (min^−1^) as the dissociation rate constant (de-phosphatization) of the trapped [^18^F]F-FAZA, as well as the total volume distribution (V_T_) and total binding potential (k_3_/k_4_) of the radiopharmaceutical. SUV measurements were derived from the static [^18^F]F-FAZA PET data set, which was acquired 120 min post-injection.

### 2.5. Statistical Analysis

Continuous and categorical variables were presented as mean (±SD) and frequency (%), respectively. Two main groups were defined as “biopsied” (i.e., cases) and “non-biopsied” (i.e., controls) based on patients’ histopathology evaluation status. Differences in variables between these two groups were assessed using non-parametric tests. eGFR was calculated from serum Cr using the CKD-EPI equation (v. 2021). Correlations between clinical parameters (the serum Cr level and eGFR) and PET-derived parameters were assessed using Spearman’s rank-order correlation. For correlations (Spearman’s) between the histopathology findings and PET-derived measurements, the pole-specific values (exactly from the biopsied kidney pole) were used for PET parameters. The statistical significance level was set at *p* < 0.05.

This study was conducted in accordance with the Declaration of Helsinki and approved by the Institutional Review Board and Ethics Committee of the University Health Network (Approval No: 17-5695; Date: 26 May 2018).

## 3. Results

Overall, 30 [^18^F]F-FAZA PET/MR procedures were acquired, including 20 post-transplant cases (with biopsy) and 10 post-transplant controls (without biopsy). Patients’ demographics and clinical information are provided in Table 1. Dynamic [^18^F]F-FAZA PET could be evaluated for 9/10 non-biopsied patients; in one patient, the dynamic PET data were corrupted due to a technical error at the acquisition level for adequate kinetic modelling. Thus, the results provided are based on 29 patients.

The recipients’ age between the biopsied (cases) and non-biopsied groups (controls) were similar, as were the recipients’ sex. There were various causes for patients’ ESKD which led ultimately to their kidney transplant, the most common ones being IgA nephropathy (*n* = 5), diabetes (*n* = 3), polycystic kidney disease (*n* = 3), hypertension (*n* = 2) and focal segmental glomerular sclerosis (*n* = 2). Other causes (each *n* = 1) included acute kidney injury, congenital renal dysplasia, medullary sponge kidney, lithium toxicity, vasculitis, Alport’s syndrome, membranoproliferative glomerulonephritis, HIV-associated, and vesicoureteral reflux. Five patients had unknown causes of ESKD. All study patients (cases and controls) had unremarkable Doppler US. Thirty-five percent of patients (*n* = 7) in the biopsied (cases) group experienced delayed graft function, compared to none in the non-biopsied group. Not surprisingly, the eGFR of the biopsied patients was markedly lower, and proteinuria was higher than in the non-biopsied patients.

**Figure 1:**
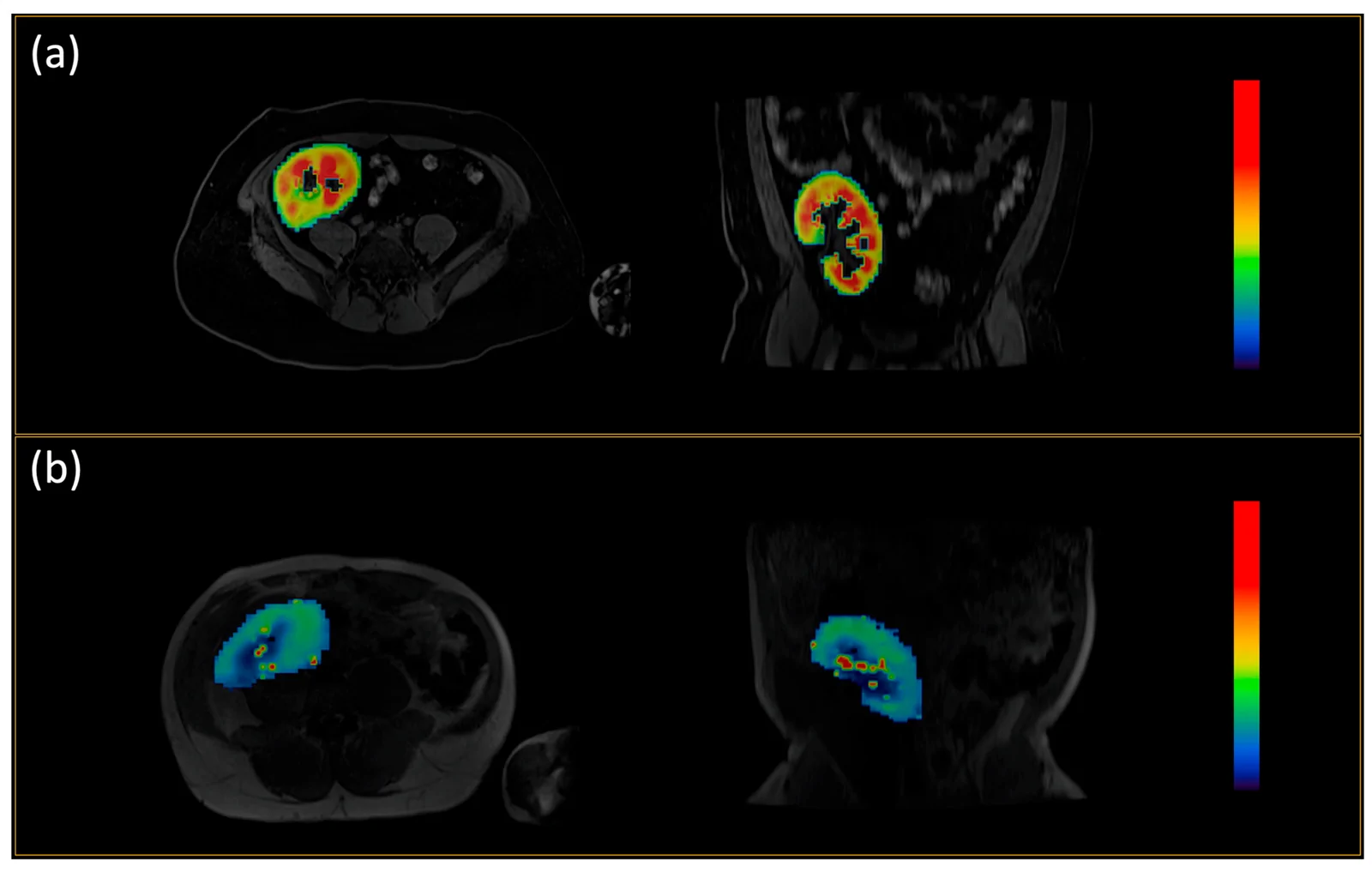
Total volume distribution (V_T_) in representative individuals with (**a**) normal kidney allograft and (**b**) kidney allograft dysfunction. As seen, the normal allograft shows high V_T_ (suggestive of normal/high microcirculation), and the dysfunctional allograft shows low V_T_ (suggestive of impaired microcirculation). Each kidney was presented in transaxial (left) and coronal (right) views.

Table 2 provides details of the [^18^F]F-FAZA PET-derived kinetic parameters. From the studied dynamic and static parameters, dynamic VT was significantly higher (*p* = 0.026) in non-biopsied patients (controls; 2.11 ± 0.55) than in malfunctioned kidneys (cases; 1.62 ± 0.45) (Figure 1).

**Figure 2:**
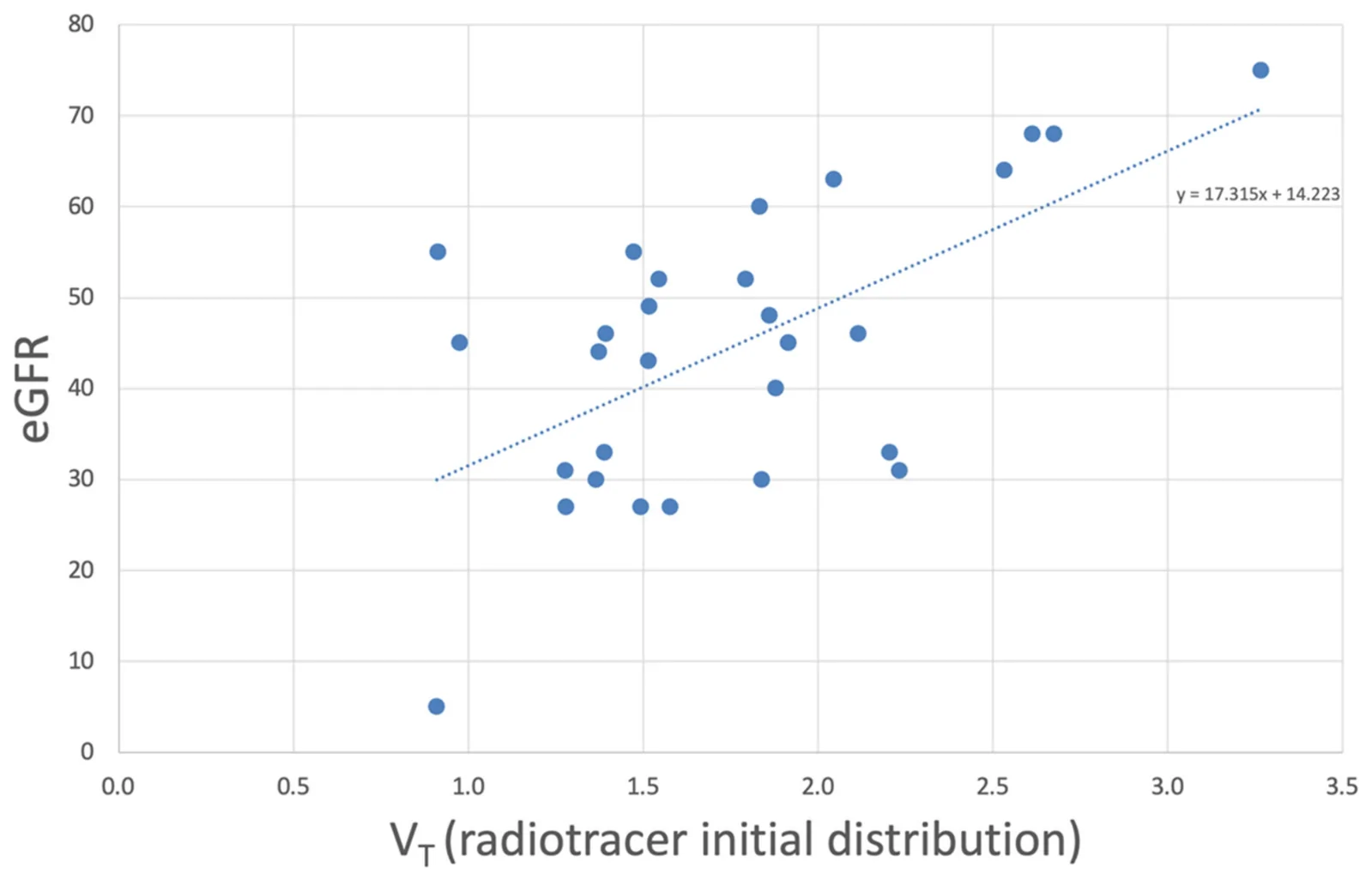
Correlation between the total volume distribution (V_T_) derived from dynamic [^18^F]F-FAZA PET and estimated glomerular filtration rate (eGFR; mL/min/1.73 m^2^) in all patients.

**Figure 3:**
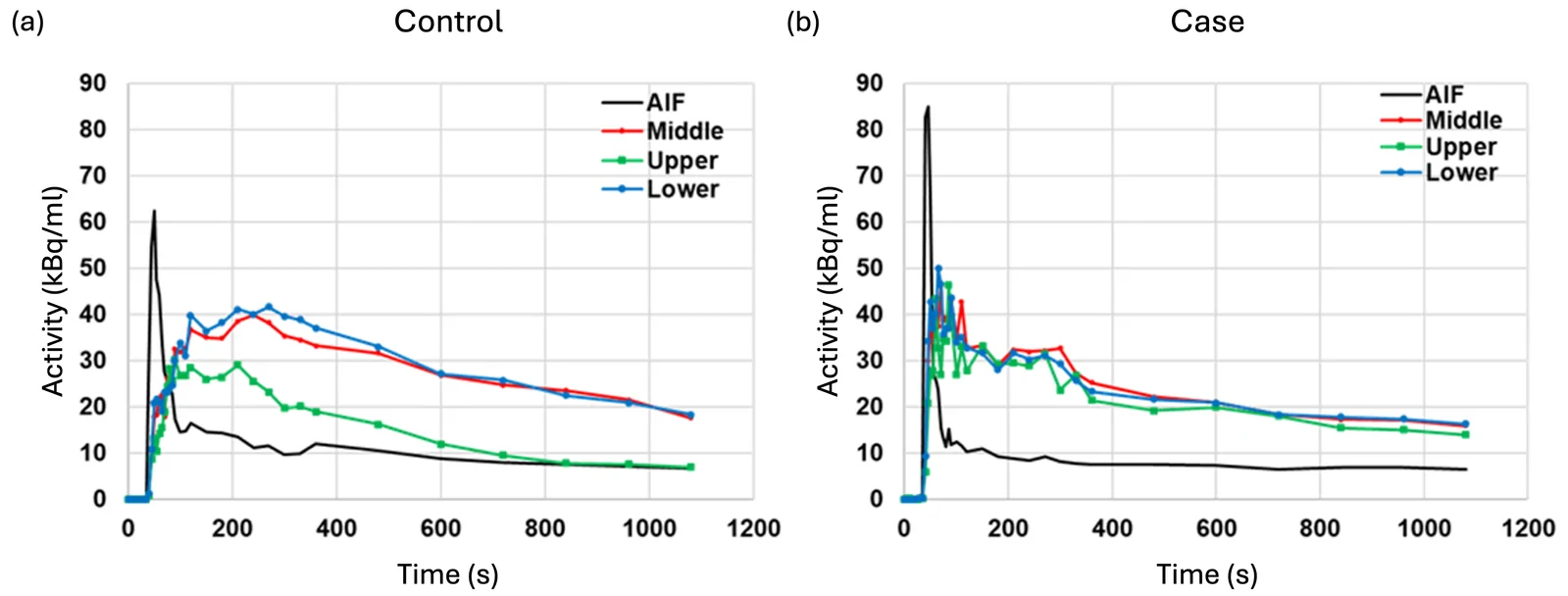
Representative [^18^F]F-FAZA PET-derived time–activity curves (TACs). Two-tissue compartment modelling is shown for (**a**) one individual from the control (non-biopsied/stable functional kidneys) group and (**b**) one individual from the case (biopsied/post-transplant malfunctioned grafts) group. AIF: arterial input function. Upper/Middle/Lower: kidney poles.

When evaluating correlations between [^18^F]F-FAZA PET parameters with serum Cr levels and eGFR (Table 3), V_T_ was again the only variable that significantly correlated with both clinical metrics (Figure 2). In addition, k_1_ and k_3_ parameters were significantly correlated with serum Cr levels. Figure 3 demonstrates representative TACs for the case and control groups.

Table 4 shows the histopathology details of the biopsied patients, based on the Banff criteria. Table 5 shows correlations between the histopathology findings and [^18^F]F-FAZA PET parameters (for this purpose, we used the pole-specific values based on the exact biopsied pole in each patient).

As seen in Table 5, a significant positive correlation was found between k_1_ and vascular fibrous intimal thickening. There was also a significant negative correlation between k_3_ and arteriolar hyalinosis. k_3_/k_4_ (binding potential) was significantly correlated with the percentage of globally sclerotic glomeruli and arteriolar hyalinosis. Meanwhile, V_T_ was significantly correlated with interstitial inflammation. Lastly, uptake on the static [^18^F]F-FAZA PET was significantly correlated with the percentage of globally sclerotic glomeruli.

## 4. Discussion

Our primary rationale was to determine the feasibility of using [^18^F]F-FAZA PET in a post-transplant patient population and to examine the association between PET-derived kinetic parameters and the metrics of kidney allograft function. Furthermore, we investigated the correlation between [^18^F]F-FAZA PET-derived parameters, as potential non-invasive functional measures of hypoxia-induced kidney allograft injury, and histological findings. For this purpose, we studied two groups: patients with stable allograft function post-transplant (non-biopsied controls) and patients with malfunctioning grafts (biopsied cases). We found that V_T_ (radiotracer total volume distribution) was higher in non-biopsied controls and was correlated with clinical metrics, serum Cr concentration and eGFR. In addition, k_1_ and k_3_ were inversely correlated with serum Cr concentration, supporting the hypothesis that these parameters have the potential to reflect kidney function.

Decreased V_T_ in [^18^F]F-FAZA dynamic PET imaging may reflect two (patho)physiologic states based on the context it is used in: (1) normoxia when tissue is well perfused and (2) tissue hypoperfusion when there is impairment in radiotracer reach [12]. Given that our static uptake (cortical SUV_mean_) was not correlated with the [^18^F]F-FAZA distribution (V_T_) in the renal cortex, we assume, similar to a previous experience with [^18^F]F-FAZA [13], that V_T_ reflected cortical microcirculation (the higher the V_T_, the better the circulation) rather than hypoxia. Aligned with this scenario, V_T_ was significantly lower in malfunctioned kidneys and showed significant correlations with Cr levels (inverse) and eGFR (positive). Notably, other parameters such as k_1_ (the rate constant of perfusion from the blood to the tissue) and k_3_ (tracer’s accumulation rate constant) were partly in alignment with V_T_ findings and showed significant inverse correlations with the serum Cr levels. Moreover, it is known that the glomerular filtration rate, which was significantly correlated with [^18^F]F-FAZA distribution (V_T_) in this study, partly aligns with renal blood flow, depending on the clinical scenario [14].

Assessing cortical microcirculation can be a powerful tool for detecting early kidney damage and may give an indication about the stages or possibly even causes of kidney impairment [15,16]. However, clinically available imaging techniques for non-invasive assessment of renal microvasculature alterations following transplant are partly limited on various levels [17]. In this at-risk population, for whom the contrast media can be problematic, clinicians mainly rely on renal Doppler ultrasound findings (e.g., resistance index, velocities), which can be influenced by a variety of extrarenal factors (e.g., age, atherosclerosis, operator handling and arterial stiffness) [6,18]. Another modality in evaluating malfunctioning grafts is an invasive kidney biopsy to establish the cause of dysfunction and rule out rejection. However, kidney lesions are heterogeneous and the small tissue evaluated may not be representative of the entire kidney. As such, having imaging modalities that capture abnormalities in the entire kidney, in a non-invasive fashion, would be a great advancement in the field.

Studies on other imaging modalities have provided ample evidence that a decrease in kidney perfusion is associated with decreased kidney function. For instance, renal blood flow was measured using the arterial spin labelling (ASL) technique in the study by Artz and colleagues. They investigated both native (healthy volunteers and CKD patients) and transplanted kidneys and showed a good correlation between cortical perfusion assessed by ASL and kidney function (eGFR) [4]. Similar findings were established in other studies on ASL, all supporting the correlation between renal impairment and decreased blood perfusion [5,7,19]. Additionally, Hueper et al. established that renal perfusion is significantly impaired in patients with delayed graft function after the kidney transplant [18]. Moreover, studies with complementary imaging techniques like intra-voxel incoherent motion (IVIM) imaging showed that kidney dysfunction is associated with reduced kidney perfusion, depicting high D* perfusion-related (microvascular blood flow) values [20].

Furthermore, hypoxia imaging using the blood oxygenation level-dependent (BOLD) technique showed a strong correlation between cortical R2* and clinical metrics, meaning higher R2* (lower oxygen bioavailability) was associated with higher serum Cr concentration, lower eGFR, chronic kidney disease and major renal events, such as ESKD [21,22,23]. Thus, our findings on V_T_, appear to be consistent with the perfusion literature, supporting the hypothesis that total volume distribution derived from dynamic [^18^F]F-FAZA PET in the renal cortex serves as a surrogate marker of tissue microcirculation rather than actual hypoxia.

Lastly, the V_T_ was significantly negatively correlated with interstitial inflammation from the kidney allograft histopathology. Interstitial inflammation, indicative of the degree of inflammation in non-scarred cortical regions, is associated with subsequent development of fibrosis and graft loss [24,25,26]. Additionally, k_1_ and k_3_ were significantly correlated with vascular fibrous intimal thickening and arteriolar hyalinosis, respectively, indicating that specific dynamic PET parameters might be indicative of a few, mostly vascular-related, underlying pathologies. Regarding the static [^18^F]F-FAZA PET, the SUV_mean_ was significantly correlated with globally sclerotic glomeruli percentage, suggesting that static PET measurements may capture nephron dropout in end-stage (sclerotic) glomeruli.

None of the studied parameters herein showed a significant level of correlation with interstitial fibrosis on biopsy. Although the kinetic parameters do not seem to predict histopathology measures of interstitial fibrosis, the thickening of the arterial intima (which leads to vascular narrowing and a decrease in tissue perfusion [24]) was significantly correlated with the measured perfusion rate, and SUV_mean_ correlated with sclerosed/fibrosed glomeruli. In this regard, it is well established that microvascular dysfunction and dropout form a vicious cycle that will eventually lead to tubular atrophy and interstitial fibrosis [5,27]. It should be mentioned that there was only one patient with severe interstitial fibrosis in the study cohort, limiting the generalisability of this finding. Also noteworthy, it was reported that changes in kidney hemodynamics could be a more reliable predictor of long-term renal damage than the tissue fibrosis itself [28], making the findings from dynamic PET imaging potentially actionable decision support measurements in the future. Thus, [^18^F]F-FAZA distribution may be providing information about renal function, complementary to what can be obtained from a biopsy and the traditional clinical assessment. Future studies are needed to determine if early defects in kidney graft perfusion predict the subsequent development of fibrosis.

Although beyond the scope of our discussion, it is worth briefly mentioning that other PET radiotracers have also been investigated to evaluate kidney diseases, one of the promising ones being radiolabelled fibroblast activation protein (FAP) inhibitor. For example, Luo and colleagues studied FAP-targeted PET and [^18^F]FDG PET in distinguishing kidney transplant rejection phenotypes [29]. They reported that FAP-targeted PET was significantly superior in identifying rejection, particularly for chronic rejection with significantly higher SUVmax. Wang et al. also showed that FAP-targeted PET uptake is significantly higher in renal parenchyma in patients with various kidney diseases, in both acute and chronic processes [30]. Uptake on FAP-targeted PET has been shown to correlate directly with histological tubulointerstitial inflammation, interstitial fibrosis, and tubular atrophy [30,31]. However, all these studies only evaluated PET measurements on standard delayed/static images, analyzing SUVs. Based on our results, future studies may benefit from adding an early dynamic phase to their PET protocol, as dynamic kinetics may reflect a different pathophysiology in the renal parenchyma compared to static PET SUV measurements.

One limitation of our study was that we used image-derived AIF for dynamic kinetic modelling, which is well established to be less accurate than arterial blood sampling. Although our methodology was a more practical approach to generate kinetic estimates and is more feasible in clinical practice as technically the most accurate non-invasive method to create an input function (when using large-calibre vessels as input, as we did, which are less susceptible to partial volume effect and spillover), our non-calibrated estimates could result in deviation from blood sampling-based kinetic calculations, such as k_1_ and V_T_ [32,33]. It is noteworthy that an important emerging technology relevant to this limitation is long axial field-of-view (LAFOV) PET, which offers substantially greater axial coverage (up to 2 m) and higher sensitivity compared with conventional PET systems [34,35]. This allows simultaneous dynamic imaging of the heart, aorta, and kidneys within a single field of view and bed position, enabling derivation of a highly reliable image-derived input function while concurrently acquiring renal time–activity curves. Previous studies have demonstrated that LAFOV-derived AIFs can achieve high accuracy relative to calibrated reference methods [36]. Thus, incorporation of LAFOV PET into future renal dynamic imaging protocols can improve the robustness and reproducibility of kinetic parameter estimation while avoiding arterial blood sampling.

This study had some other limitations as well. Most importantly, we investigated a relatively small patient cohort. As expected, this could considerably limit the statistical power and generalisability of our findings. Also, we explored multiple correlations despite the limited sample size, increasing the possibility of type I errors. However, our small sample size was because of the pilot nature of our investigation, before any larger-scale trial, which is methodologically necessary for an initial assessment of a novel hypothesis. Larger, adequately powered studies with pre-specified correlative hypotheses are needed to further assess the reported associations in our results. Additionally, it is noteworthy that we recruited age- and sex-matched cases as well as controls in our cohort, limiting the known confounding effect of patients’ demographics [7]. Another limitation was that the [^18^F]F-FAZA PET-derived parameters could only be correlated with histological findings in a subgroup of individuals, those who had impaired kidney function, since not all patients required biopsies. Also, underlying pathologies that lead to impaired kidney function were heterogeneous. Lastly, analyses of dynamic [^18^F]F-FAZA PET and feature extraction were performed semi-automatically, making the findings prone to operator-dependency.

## 5. Conclusions

Our findings in a cohort of kidney transplant recipients showed that the non-invasive assessment of renal microcirculation using dynamic [^18^F]F-FAZA PET is potentially feasible. The dynamic [^18^F]F-FAZA PET-derived parameters showed strong correlations with kidney function. Also, notable correlations were found with different histopathology findings. Static [^18^F]F-FAZA PET-derived parameters showed complementary findings with hypoxia-related end-stage histopathological indicators. Thus, dynamic [^18^F]F-FAZA PET-imaging may enable non-invasive detection of renal injury in transplant recipients, potentially providing information about histopathology lesions and kidney function, complementary to what can be obtained from a biopsy and the traditional clinical assessment.

## Figures and Tables

**Table 1:** Patients’ demographics.

	Whole Cohort (*n* = 29)		Biopsied (Cases/*n* = 20)	Non-Biopsied (Controls/*n* = 9)
	Mean (±SD)	Frequency (%)		
Recipient age	53.41 (±13.82)		53.80 (±14.83)	52.56 (±12.06)
Donor age	49.26 (±13.64)		52.72 (±11.41)	42.33 (±15.72)
Recipient sex (female)		15 (52%)	10 (50%)	5 (55%)
Donor sex (female)		13 * (46%)	10 * (53%)	3 (33%)
Donor type (living)		13 (45%)	9 (45%)	4 (44%)
Transplant number - 1 - 2 - 3		26 (90%) 2 (7%) 1 (3%)	18 (90%) 1 (5%) 1 (5%)	8 (89%) 1 (11%) 0 (0%)
History of diabetes		6 (21%)	4 (20%)	2 (22%)
Delayed graft function		7 (24%)	7 (35%)	0 (0%)
eGFR at the time of scan (mL/min/1.73 m^2^)	44.55 (±15.72)		39.20 (±14.49)	56.44 (±11.63)
Serum creatinine (µmol/L)	173.97 (±160.64)		199.45 (±188.92)	117.33 (±17.88)
Proteinuria (mg/mmol creatinine)	28.27 (±85.27)		40.02 (±104.33)	6.06 (±10.20)
Transplant to scan interval (days)	832.72 (±629.51)		681.45 (±644.90)	1168.89 (±463.57)
Graft loss during follow-up		3 (10%)	3 (15%)	0 (0%)

* Information for one patient was missing.

**Table 2:** Details of the [^18^F]F-FAZA PET-derived parameters.

	Mean (±SD)	Non-Biopsied	Biopsied	*p*-Value
k_1_ [mL/ccm/min]	1.96 (±0.93)	2.45 (±1.06)	1.74 (±0.79)	0.105
k_3_ [1/min]	0.75 (±0.68)	0.92 (±0.75)	0.68 (±0.66)	0.594
k_4_ [1/min]	1.17 (±0.64)	1.34 (±0.59)	1.10 (±0.66)	0.340
k_3_/k_4_	0.76 (±0.67)	0.75 (±0.66)	0.76 (±0.69)	0.945
V_T_ [mL/ccm]	1.77 (±0.53)	2.11 (±0.55)	1.62 (±0.45)	0.026 *
SUV_mean_	5.04 (±0.84)	4.83 (±0.88)	5.13 (±0.83)	0.501

* Statistically significant.

**Table 3:** Correlations between [^18^F]F-FAZA PET parameters and laboratory metrics.

		K_1_	K_3_	K_3_/K_4_	V_T_	SUV_mean_
Serum creatinine level	r_s_	−0.352	−0.401	0.013	−0.446	0.119
	*p*-value	0.031 *	0.016 *	0.473	0.008 *	0.270
Estimated glomerular filtration rate	r_s_	0.238	0.267	−0.119	0.467	−0.135
	*p*-value	0.107	0.081	0.270	0.005 *	0.242

* Statistically significant.

**Table 4:** Histopathology details of the biopsied patients, based on the Banff criteria.

Patient ID	*# Sclerosed Glomeruli*	*%gsg*	*g*	*mm*	*cg*	*i*	*t*	*ptc*	*ci*	*ct*	*v*	*ah*	*cv*
**1**	2	8.3	0	0	0	0	0	0	1	1	0	1	0
**2**	0	0.0	1	1	1	0	0	1	1	1	0	3	2
**3**	2	9.1	0	1	0	1	1	0	2	2	0	1	1
**4**	3	10.7	1	1	0	0	1	0	2	2	0	2	2
**5**	2	22.2	0	0	0	1	1	0	1	1	0	0	0
**6**	1	5.6	0	1	0	0	1	0	0	0	0	1	2
**7**	0	0.0	0	0	1	0	0	0	0	0	0	0	2
**8**	3	10.0	0	0	0	0	0	0	0	0	0	2	0
**9**	2	5.9	0	0	0	2	3	0	3	3	0	3	3
**10**	3	9.7	1	0	0	3	3	1	2	2	0	1	1
**11**	0	0.0	2	2	0	0	0	2	1	1	0	3	3
**12**	0	0.0	0	0	0	0	1	0	1	1	0	2	2
**13**	0	0.0	1	0	0	0	1	1	1	1	0	0	0
**14**	9	25.0	0	1	0	0	0	1	1	1	0	1	0
**15**	0	0.0	0	0	0	0	0	0	1	1	0	0	0
**16**	4	8.9	3	2	3	0	0	1	2	2	0	1	2
**17**	0	0.0	0	0	0	1	0	1	1	1	0	1	1
**18**	4	28.6	2	1	0	0	1	3	2	2	0	2	2
**19**	2	9.1	0	1	1	0	0	0	1	1	0	1	1

%gsg, globally sclerotic glomeruli (%); g, glomerulitis; mm, mesangial matrix expansion; cg, chronic glomerulopathy; i, interstitial inflammation; t, tubulitis; ptc, peritubular capillaritis; ci, interstitial fibrosis; ct, tubular atrophy; v, endarteritis; ah, arteriolar hyalinosis; cv, vascular fibrous intimal thickening.

**Table 5:** Correlations between [^18^F]F-FAZA PET parameters and histopathology findings in the biopsied patients (cases).

		K_1_	K_3_	K_3_/K_4_	V_T_	SUV_mean_
Globally sclerotic glomeruli (%)	r_s_	−0.220	0.164	0.533	−0.059	−0.517
	*p*-value	0.183	0.251	**0.009** *	0.405	0.012 *
Glomerulitis	r_s_	0.158	−0.104	0.094	0.115	0.214
	*p*-value	0.260	0.337	0.351	0.319	0.189
Mesangial matrix expansion	r_s_	−0.013	−0.133	−0.207	0.242	−0.076
	*p*-value	0.478	0.293	0.198	0.159	0.378
Chronic glomerulopathy	r_s_	0.279	0.134	0.083	0.185	0.084
	*p*-value	0.123	0.292	0.368	0.224	0.366
Interstitial inflammation	r_s_	0.109	0.189	0.196	−0.432	−0.171
	*p*-value	0.328	0.219	0.211	**0.032** *	0.241
Tubulitis	r_s_	0.136	0.237	0.175	−0.147	−0.149
	*p*-value	0.289	0.164	0.237	0.275	0.272
Peritubular capillaritis	r_s_	−0.171	0.069	0.172	−0.075	0.271
	*p*-value	0.242	0.390	0.241	0.381	0.131
Interstitial fibrosis	r_s_	0.100	−0.101	0.219	−0.173	0.025
	*p*-value	0.342	0.340	0.184	0.240	0.460
Tubular atrophy	r_s_	0.100	−0.101	0.219	−0.173	0.025
	*p*-value	0.342	0.340	0.184	0.240	0.460
Arteriolar hyalinosis	r_s_	0.070	−0.566	−0.416	0.269	−0.178
	*p*-value	0.388	**0.006** *	**0.038** *	0.133	0.233
Vascular fibrous intimal thickening	r_s_	0.422	−0.112	−0.302	0.271	0.291
	*p*-value	**0.036** *	0.324	0.105	0.131	0.113
Percentage fibrosis *	r_s_	0.030	−0.059	0.209	−0.193	−0.033
	*p*-value	0.452	0.406	0.195	0.214	0.447

* This continuous histopathological parameter is a non-graded addition to the Banff-based ordinal metrics, being measured by an expert renal pathologist.

## Data Availability

All data are available with the corresponding author and can be provided following a reasonable request.

## Abbreviations

[^18^F]F-FAZA	^18^F-labelled fluoroazomycinarabinoside
ESKD	End-stage kidney disease
Cr	Creatinine
eGFR	Estimated glomerular filtration rate
VOI	Volumes of interest
TAC	Time–activity curves
2TC	2-tissue compartment
V_T_	Volume distribution
IVIM	Intra-voxel incoherent motion
BOLD	Blood oxygenation level-dependent
